# Social cognitive determinants of HIV voluntary counselling and testing uptake among married individuals in Dar es Salaam Tanzania: Theory of Planned Behaviour (TPB)

**DOI:** 10.1186/s12889-015-1545-4

**Published:** 2015-03-04

**Authors:** Sally M Mtenga, Amon Exavery, Deodatus Kakoko, Eveline Geubbels

**Affiliations:** Ifakara Health Institute, P.O. Box 78373, Dar Es Salaam, Tanzania; Muhimbili Department of Health Science, (MUHAS), P.O. Box 65015, Dar Es Salaam, Tanzania

**Keywords:** HIV/AIDS, HCT uptake, Married individuals, TPB, Social cognitive, Dar es Salaam, Tanzania

## Abstract

**Background:**

Cumulative evidence indicates increasing HIV infection among married individuals. Voluntary Counselling and Testing for HIV (HCT) is known to be an effective intervention to induce safer sex behaviour and access to early treatment, care and support among married individuals, which are important for HIV prevention. In this context, knowledge of factors associated with HCT uptake among married individuals is critical in promoting the use of the services. This study therefore intended to identify the social cognitive factors associated with acceptance of HCT among married individuals.

**Methods:**

In a cross-sectional analytical study face to face questionnaires were administered among 200 randomly selected married individuals in Kinondoni district, Dar es Salaam Tanzania. The questionnaire included self-reported HCT, socio-demographic variables and social cognitive variables (attitude, subjective norms, perceived control and perceived risk). Logistic regression was used to identify the independent association of social cognitive predictors of HCT among married individuals.

**Results:**

Nearly half (42%) of the respondents had never had HCT. Of the social cognitive constructs, the strongest predictor of HCT uptake was attitude (OR per additional score point = 1.07, 95% CI 1.04-1.10) followed by perceived behavioural control (OR = 1.04, 95% CI 1.02-1.06). Subjective norm and perceived risk were not associated with HCT uptake.

**Conclusion:**

Public health interventions targeting married individuals should be designed to enhance their positive attitude towards HCT and empower them to overcome barriers to the use of the services.

## Background

More evidence in sub-Saharan Africa (SSA) indicates the shift of HIV infections being primarily contracted by people who are in casual relationships to those in stable relationships [1-3]. Specific evidence indicates that up to 60% of new infections in SSA are acquired within marriage or cohabitation [4,5]. Tanzania is among the countries in SSA in which the overall HIV prevalence has been reported to decline [6], however, the downtrend is much more apparent among young people (15–24 years) as compared to adults, and in unmarried individuals as compared to married couples [6,7]. About 5% of married or cohabitating partners are reported to be in HIV discordant relationships [6]. Despite this high prevalence, knowledge of one’s HIV status remains low in SSA [8].

Evidence indicates that HIV counselling and testing (HCT) is effective in reducing the risk of HIV transmission among married partners. For instance, access to HCT has been reported to contribute in reducing the number of sexual partners and increasing condom use with spouses for both HIV-negative and HIV-positive respondents [9-12]. HCT also provides opportunity for implementing an evidence-based treatment model for prevention, promoted as *Testing* and *Treatment* for every HIV-infected person as soon as diagnosis is done [13,14]. The Ministry of Health and Social Welfare (MoHSW) in Tanzania is currently providing various modes of HIV testing with a goal of expanding access to HCT. This includes Client Initiated and Counselling and Testing (CICT), Provider Initiated Counselling and Testing (PICT), and antenatal HCT services. Mobile HCT services and home based HCT services have also been implemented by different stakeholders in health, and as part of targeting special groups. Couple centred HCT is also provided within the HCT service delivery system [15,16].

Despite ample evidence on the significance of HCT and the widespread HCT services, most married individuals are unaware of their sero-status [17-19] and live in speculation, bearing some misconceptions of their partner’s HIV status [19]. For instance, a recent study in Kenya revealed that about 83.6% of HIV-infected Kenyans living in married or cohabitating couples neither partner knew their HIV status [20]. In some cases, married men have been relying on their spouse’s HIV status to deduce their own [21]. In Tanzania, only 29.8% of married men and 32% of married women received results from a HIV test taken in the past 12 months prior to survey [22] and only 32% of male spouses have ever had an HIV test in their lifetime [6].

Beliefs, contextual and social barriers have been associated with HCT uptake among adults. These include: a belief that discordance cannot exist within couples [23], education level and age [24], treatment availability [25], fear of being denied sexual rights [26], social stigma, lack of partner support and fear of knowing HIV status [27,28].

Although numerous researchers have investigated the barriers to HCT uptake among adults with the scaling-up of these services, to date, very few studies have used theoretical model in explaining HCT uptake among married individuals. To the best of our knowledge, this is the first study to apply the TPB in a population of people in steady relations. Guided by the theory of Planned Behaviour (TPB) this study contributes to the existing knowledge by examining the social cognitive correlates of HCT uptake among married individuals.

### Theory of Planned Behaviour [TPB]

The TPB [29] is a social cognitive model, which is an extension of the Theory of Reasoned Action (TRA) [29-32]. The model intends to predict and explain individuals’ behaviours in relation to various beliefs.

The TRA is composed of two constructs; attitude and subjective norms, considered as immediate determinants of behaviour performance [30]. TRA assumes that people are rational actors. Later, Ajzen and Madden proposed the TPB in order to also account for behaviors that were not under volitional control [33]. In this respect, another construct ‘perceived behaviour control’ was added in the model. In the TPB, attitude, subjective norms and perceived behaviour control are the core constructs on the same level of predicting behavior and assumed to combine multiplicatively. To increase the prediction power of the model, Ajzen [33] recommended an addition of other constructs in the model after the core TPB constructs have been considered. In the same reasoning, this study added ‘perceived risk’ of HIV, a construct derived from the Health Belief Model (HBM) [34] due to engagement in risk behaviours among married individuals in Tanzania [6].

The TPB has been widely tested and validated in various social cultural contexts including Tanzania [34-37], and was considered relevant for this study as it appreciates volitional and non volitional behaviours with an assumption that partner’s self beliefs and society including spouses may both play part in decisions to seek HCT.

## Methods

### Study design and settings

A cross sectional survey was conducted between May and June, 2010 among married individuals living in Kinondoni district in Dar es Salaam municipality, Tanzania. Dar es Salaam is located on the eastern coast of Tanzania and is a centre of commercial and social activities. Kinondoni is a multi-ethnic district with a population of 1,083,913 [38], located in the northern part of Dar es Salaam and has well established HIV prevention programmes, including HCT services. The HIV prevalence in Dar es Salaam in the years prior to conducting the study was 10.6% for women and 7.9% for men [6].

### Study population and sampling procedure

The study involved married individuals aged 18 years and above. We only considered married individuals living with their spouses at the time of study and selected the age of 18 years and above since this is the legal age for marriage in Tanzania [39]. To avoid a situation in which participants would not freely give their views, in each couple we requested only one of the spouses to participate in the interview alternating between the female and male spouse. With nine independent variables to be tested in multivariable modeling, 10 events required per variable and an expected HCT uptake of 45%, the resulting sample size was 200. We employed multistage sampling to select two streets from the district, one located at the periphery and another street at the centre of the district. Using simple random sampling we drew 200 households from the streets’ residence register books.

### Data collection and instrument

Face to face interviews were conducted with eligible married individuals using a pre-tested questionnaire. The questions were administered in *Kiswahili,* the national language which is widely spoken and understood in Tanzania. This questionnaire had been translated from English to Kiswahili and back-translated to ensure validity of the language used. The interviews were conducted by trained and experienced research assistants in a location within the household preferred by the respondent. Participants were asked about their social demographic information (age, education, occupation, religion, duration of marriage, type of marriage, location) and their opinions with regards to the theoretical constructs under study (attitude, perceived behaviour control, subjective norms and perceived risk).

### Measurements

In this study we defined married individuals as those (a man and a woman) who were in marital or cohabiting relations. Reported use of complete HCT by individuals was the main dependent variable. Complete HCT was defined as having gone through all three of the components of HCT, i.e. pre-test counselling, HIV test and receiving results following post-test counselling [40]. Individuals who reported that they received all the three HCT components were considered to have achieved a complete HCT and if an individuals reported to have received only one or two of the components were considered to have received incomplete HCT.

#### Theoretical constructs

Constructs (attitude, subjective norm, perceived behavioural control) from the TPB and one construct from the HBM (perceived risk) were the main variables of interest in this study. The items used to measure theoretical construct variables were adopted from previously used items in a study on HCT behaviour among teachers in Tanzania [35]. To increase relevance to this study’s population, in the subjective norms construct we added ‘spouse’, ‘children’ and ‘in-laws’ to the significant other’s list. We measured each variable for the theoretical model using a 5 item Likert type of scale consisting of various items. Under each of the valuation scores, we asked respondents to indicate their opinion about the statements by assigning a degree of their opinion ranging from number 1 to 5. The following is a break-down of each of the constructs identified from the TPB &Health Belief Model:

#### Attitude

*We assessed attitude by asking respondents to indicate the extent to which they think that HCT is likely to provide certain benefits (behaviour beliefs) and the extent to which they think that if they use the HCT the indicated benefits are going to occur (belief outcome). We used items of ‘behavioural beliefs’ (individuals’ beliefs about the outcomes of using HCT) together with those of ‘outcome evaluation’ (belief outcomes) to compose attitude. ‘Behaviour beliefs’ comprised the following statement; please tell me the extent to which you think your use of HCT services is likely to: avert your chances of HIV infection, make it possible for you to avoid transmitting HIV to a spouse, result in a happy life if test results are negative, help you plan confidently for their future and facilitate your seeking of anti-retroviral therapy (ARVs) if HIV test results are positive. Respondents were asked to indicate their opinion to the statements based on valuation of the following items ranging from 1 = Not likely at all, 2 = unlikely, 3 = not sure, 4 = likely, 5 = very likely. ‘Belief outcome’ was assessed by asking the respondents to rate how good or bad they thought that visiting a HCT centre would be important. The evaluation scores ranged from 1 = extremely bad, 2 = bad, 3 = Not sure, 4 = good, 5 = extremely good. To calculate the attitude scale, each item of ‘behavioral belief’ score was multiplied with items of ‘outcome evaluation’ score, to calculate the attitude scale. And later the summation of the total scores was generated. Number ‘1’ indicated a least favourable attitude and number ‘5’ more favourable attitude towards HCT services. Such that the higher the scores the more favourable attitude towards HCT. The same process was applied in other social cognitive variables.*

#### Subjective norm

*In this study, subjective norm denoted a respondent’s perception of significant others’ opinions on whether one should or should not use the HCT. We asked respondents to indicate the extent to which they thought their spouses/partner, in-laws, children, relatives, religious leaders, best friend, counsellors, doctors, parents, best man or best lady for your wedding and neighbours would be likely to endorse their use of HCT (normative beliefs). A five Likert scale comprised of 1 = not likely at all, 2 = unlikely, 3 = not sure, 4 = likely, 5 = very likely, was used to assess respondents opinions. Respondents were also asked to rate the extent to which they thought it was important for them to comply with the wishes of their referents (Motivation to comply). Respondent’s opinions were assessed using the following items: 1 = not important at all, 2 = not important, 3 = not sure, 4 = important, 5 = very important.*

#### Perceived behavioural control

*Perceived behaviour control reflected the respondent’s perception with regards to their capacity to overcome barriers for using HCT. Respondents were asked to indicate their opinion on the extent to which the following items would deter them from using the HCT services: cost related to HCT testing was high, they will die more quickly if tested positive for HIV, HCT service providers cannot keep the test results confidential, you would be stigmatized if you are known or suspected to be HIV-infected (control beliefs). The scale measurement comprised of 1 = not at all, 2 = not, 3 = not sure, 4 = much, 5 = very much. Then, we asked respondents to rate how difficult or easy was it for them to use the HCT services next time they go for a health facility if the above barrier items under ‘control beliefs’ were true (power of control). The scale measurement comprised of 1 = very difficult, 2 = difficult, 3 = not sure, 4 = easy, 5 = very easy.*

#### Perceived risk

*We measured perceived risk by asking respondents to indicate the extent to which they considered AIDS as a threatening disease. The scale items comprised of 1 = not likely at all, 2 = unlikely, 3 = not sure, 4 = likely, 5 = very likely. Then, respondents were asked to indicate the extent to which they disagreed or agreed with the following statements; ‘AIDS is a dangerous disease’, AIDS is a dangerous disease in Tanzania’, ‘AIDS is a dangerous disease in your communities’, AIDS is a dangerous disease among married individuals’, ‘AIDS is a dangerous disease to you. The scale items comprised of 1 = strongly disagree, 2 = disagree, 3 = not sure, 4 = agree 5 = strongly agree.*

### Data analysis

We performed descriptive analyses for all the categorical variables using frequencies and percentages. We then conducted bivariable analysis to assess the extent of HCT uptake by each of the independent variables. We used Chi-square test to test the degree of association between HCT and each of the categorical independent variables. For continuous predictors, specifically attitude, subjective norm, perceived behaviour control, and perceived risk, *t*-test was used to compare their mean scores across categories of HCT uptake. Finally, we performed multivariable analysis using logistic regression to assess independent factors associated with HCT uptake among married individuals in the study area. Selection of variables for multivariable analysis was based on their ability of each one to improve the model. This was tested using the log-likelihood ratio test [41] on consecutive nested models. However, the social cognitive variables – attitude, perceived behaviour control, subjective norms and perceived risk – were automatically retained in the multivariable model because they were the main focus of the study. The model was also checked for statistical interaction. We set the level of significance at 5%.

### Ethics

Written informed consents were sought from the participants prior to interviews. We did not interview a husband and a wife from the same household. Prior to interviewing a spouse of a head of household, the household head was asked for consent for the interviews to be conducted in the household. In the context where the respondent was the head of the household, s/he only provided own consent to participate in the study. The Ifakara Health Institute’s Institutional Review Board (IRB) approved the study.

## Results

### Background characteristics of the study participants

A total of 200 married individuals participated in the study (97.4% response rate). As presented in Table 1, the studied group constituted of 98 (49%) males and 102 (51%) females. Less than half of the respondents (42%) had never used HCT. The majority of respondents were within the age range of 35 to 44 years (36%) followed by those of age 25 to 34 years (31.5%). Half (50.5%) of the respondents reported to have been married for 7+ years followed by those who had been married for 4 to 6 years (28.5%) and those who had been married for 1 to 3 years (21%). Furthermore, monogamous union accounted for the majority (86%) of the respondents. With respect to education two-thirds of the respondents reported to have attained a secondary or higher education level, while the rest had primary education or less. The majority of the study participants were Muslims (68.5%). About (46.5%) of the respondents were employed while those with self employment were 40.5% and the remaining 13% reported to have no job (Table 1).

**Table 1:** **Social demographic characteristics of the respondents in Kinondoni district of Dar es Salaam region in Tanzania, 2010**

	**Number of respondents (n)**	**Percent (%)**
**OVERALL**	200	100.0
**Age group (years)**		
18-24	25	12.5
25-34	63	31.5
35-44	72	36.0
45+	40	20.0
**Gender**		
Male	98	49.0
Female	102	51.0
**Religion**		
Christian	63	31.5
Muslim	137	68.5
**Marriage type**		
Polygamous	28	14.0
Monogamous	172	86.0
**Duration of marriage (years)**		
1-3	42	21.0
4-6	57	28.5
7+	101	50.5
**Education**		
Primary or below	67	33.5
Secondary+	133	66.5
**Occupation**		
No job	26	13.0
Employed	93	46.5
Self employed	81	40.5
**Location**		
Mwananyamala	120	60.0
Kibamba	80	40.0

### HCT uptake by background characteristics

Table 2 shows bivariate analysis of factors associated with HCT uptake. Overall 58% of all respondents had used HCT. This proportion varied significantly by some characteristics of the respondents. The proportion of respondents who had used HCT was significantly higher among Christians when compared to Muslims (73% versus 51.1%, P = 0.004). Those with secondary school education were significantly more likely to use the HCT than those with primary education or below (66.9% versus 40.3%, P < 0.001).

**Table 2:** **Percentage distribution of HCT uptake by social demographic characteristics of the respondents in Kinondoni district of Dar es Salaam region in Tanzania, 2010 (n = 200)**

	**% HCT uptake**	**P-value**
**OVERALL**	58.0	−
**Age group (years)**		
18-24	56.0	0.190
25-34	66.7	
35-44	58.3	
45+	45.0	
**Gender**		
Male	52.0	0.094
Female	63.7	
**Religion**		
Christian	73.0	0.004
Muslim	51.1	
**Marriage type**		
Polygamous	46.4	0.181
Monogamous	59.9	
**Duration of marriage (years)**		
1-3	47.6	0.163
4-6	66.7	
7+	57.4	
**Education**		
Primary or below	40.3	<0.001
Secondary+	66.9	
**Occupation**		
No job	46.2	0.600
Employed	66.7	
Self employed	51.9	
**Location**		
Mwananyamala	57.5	0.861
Kibamba	58.8	

Table 3 describes mean score of each of the TPB and HBM variables - attitude, subjective norms, perceived behaviour control and perceived risk by HCT uptake. Attitude, subjective norms and perceived behaviour control were significantly associated with HCT uptake, whereby as mean score of the TPB variables increased, HCT uptake increases as well. Mean attitude was significantly higher among those who reported HCT uptake compared to those who did not (113.8 versus 97.4, P < 0.001). Perceived behavioural control mean score was significantly higher among those who reported HCT uptake compared to those who did not (51.8 versus 36.3, P < 0.001). Perceived risk mean score was not significantly higher among those who reported HCT uptake compared to those who did not (9.8 versus 10.0, P = 0.380). Mean subjective norm was significantly higher among those who reported HCT use compared to those who did not use HCT (187.9 versus 145.8, P < 0.001).

**Table 3:** **Mean scores of social cognitive factors by HCT uptake in Kinondoni district of Dar es Salaam region in Tanzania, 2010 (n = 200)**

**socio cognitive factors**	**HCT uptake**	**Mean score**	**95% CI**	**P-value**
Attitude	Yes	113.8	111.36-116.26	<0.0001
	No	97.4	93.70-100.99	
Perceived behaviour control	Yes	51.8	48.25-55.32	<0.0001
	No	36.3	32.25-40.34	
Subjective norm	Yes	187.9	172.35-203.46	0.0001
	No	145.8	132.71-158.86	
Perceived risk	Yes	9.8	9.37-10.13	0.3803
	No	10.0	9.50-10.58	

### Predictors of HCT uptake

Table 4 shows results of the multivariable logistic regression analysis of variables associated with HCT uptake. The strongest social cognitive predictor of HCT uptake was attitude, whereby its increase by one score increased the odds of HCT uptake by 7% (OR = 1.07, 95% CI 1.04-1.10). Similarly, a one score increase in perceived behavioural control resulted in 4% increase in the odds of HCT uptake (OR = 1.04, 95% CI 1.02-1.06). The effect of subjective norm on HCT uptake was at the borderline (OR = 1.01, 95% CI 0.999-1.02). Though participants who were married four years and more were more than three times as likely to have had HCT, this effect was not statistically significant. None of the other socio-demographic variables were associated with HCT uptake.

**Table 4:** **Multivariable logistic regression analysis of factors associated with HCT uptake among married individuals in Kinondoni district of Dar es Salaam region in Tanzania, 2010 (n = 200)**

**Covariate**	**Adjusted odds ratio (AOR)**	**95% confidence interval (CI)**
**Perceived risk**	1.03	0.87 − 1.20
**Attitude**	1.07***	1.04 − 1.10
**Subjective norm**	1.01	1.00 − 1.02
**Perceived behaviour control**	1.04***	1.02 − 1.06
**Age group (years)**		
18-24 (Ref)	1.00	−
25-34	0.89	0.21 − 3.66
35-44	0.58	0.11 − 3.17
45+	0.61	0.10 − 3.82
**Gender**		
Male (Ref)	1.00	−
Female	1.65	0.74 − 3.68
**Religion**		
Christian (Ref)	1.00	−
Muslim	0.54	0.23 − 1.32
**Marriage type**		
Polygamous (Ref)	1.00	−
Monogamous	1.66	0.52 − 5.26
**Marriage duration (years)**		
1-3 (Ref)	1.00	−
4-6	3.54	0.96 − 13.00
7+	3.41	0.83 − 13.96
**Education**		
Primary or below (Ref)	1.00	−
Secondary+	1.84	0.80 − 4.25

***P < 0.001; Ref = Reference/baseline category; Hosmer-Lemeshow goodness-of-fit test, p = 0.791.

## Discussion

Guided by the TPB model, in this study, we identified the social cognitive factors associated with HCT uptake among married individuals. Attitude towards HCT and perceived behavior control were the main TPB constructs that contribute significantly to HCT uptake among married individuals in the study area. These findings are similar with previous studies conducted in Ethiopia and Tanzania. A study done in Ethiopia found that among the high school teachers, attitude accounted for a significant proportion of variability for intention to use HCT services followed by PBC [36]. Another study in Ethiopia among the health professionals found that attitude was one of the significant predictors for the intention to use HCT services [42]. In Tanzania among primary school teachers, attitude and PBC contributed much variance in the intention to use HCT, followed by subjective norms [35].

As indicated in the present study, attitude was the most important predictor of HCT uptake than other TPB constructs. This implies that attitude towards HCT plays an important role in the use of HCT services and that individuals within marital unions are likely to use the HCT services if they develop a favourable opinion about the values of HCT services. The reason for this may be that married individuals may have feelings that they are safer since they live in dyadic committed relationship. They may also have optimistic bias in terms of perception about their susceptibility to HIV infection such that decision to use the HCT services would be most likely to be influenced by what they perceive to be the benefits of their participation in HCT services rather than their susceptibility to HIV infection. This may also be a possible explanation on the non-significant association of perceived risk and HCT uptake among married individuals as found in the present study. A favourable opinion towards HCT services among married individuals could be attained if the benefits and values of HCT services such as ability to avert HIV transmission within and outside the marriage, early access to treatment and support programmes are integrated in the HCT promotion messages targeting married individuals rather than simply instructing spouses to go for HCT.

We found perceived behavioral control to be another strong determinant for HCT uptake among married individuals. Perceived behavioral control was shown to be a significant predictor of the intention to use HCT among teachers in Tanzania [35] and in Ethiopia [36] but not among the health professional in Ethiopia [42]. Our finding implies that use of HCT by married individuals would also require a sense of confidence to overcome any perceived barriers to the use of the services. In general, perceived behavioural control indicates that the more resources and opportunities individuals believe they possess, and the fewer obstacles or impediments they anticipate, the greater should be their perceived behaviour control over the barriers to behaviour performance [29]. In order to increase a sense of behaviour control among spouses it may be important that the spouses are encouraged to control feelings related to fear of stigma, discrimination and gender-based violence following test results [43,44]. In non–theory based studies it was found that possessing negotiation skills [43] and inter-spousal communication [45,46] have the potential to induce the use of other health services among spouses.

The subjective norm construct, which was not a significant predictor of HCT uptake in this study, was found to be the strongest predictor of intention for HCT among the health professionals in Ethiopia [42]. This discrepancy may indicate that social cognitive constructs have different patterns of influence among different population groups depending on their contextual background. Hence, focused interventions based on the evidence from specific contexts are likely to improve the HCT uptake. According to the TPB, where discrepancy of results is observed in various contexts, the explanation would be the difference in behaviour, the target population and circumstances in which behaviour occurs [31].

The findings that subjective norm and perceived risk were not associated with HCT uptake, might be explained by married individuals’ feelings of being safe in their marriage. This may influence their general perception of how risky HIV is at different levels. Married individuals might also feel that for the decision to use the HCT services they may not need an influence of significant others.

### Limitations of the study

The study relied on self-reports by the married individuals. This may have led to reporting bias. However, this was minimized because during the informed consent processes spouses were assessed to check whether they understood the essence of the study and they committed to provide information relevant for the study. We also couples from different households which could have enhanced the reliability of the information. Also the completeness of the data set suggests that this may not have been a significant limiting factor.

No causal inferences may be drawn from these findings because of the cross-sectional design of our study. We thus recommend future studies to use powerful designs with which temporality in the association is possible to assess.

## Conclusion

Our results clearly suggest that attitude and perceived behaviour control are the social cognitive factors that determine HCT uptake among married individuals. Subjective norms and perceived risk of contracting HIV had no influence on the use of HCT among the study participants. It is thus recommended that public health programmes should be designed to enable married couples to attain a positive attitude towards the HCT services. One possible way is for the educational messages to clearly promote the benefits of HCT services instead of instructing couples to use the services. Furthermore, education programmes should be designed to empower individuals to attain a sense of capacity to overcome barriers to the use of the HCT services. This need to be accompanied by providing a supportive environment for couples to undergo counselling and testing, which may include free HCT services, couple friendly HCT, antiretroviral treatment for the infected partners, addressing stigma related to HIV testing and enhancing social support.
